# *In Vitro* Effect of Cell Phone Radiation on Motility, DNA Fragmentation and *Clusterin* Gene Expression in Human Sperm

**DOI:** 10.22074/ijfs.2015.4217

**Published:** 2015-04-21

**Authors:** Adel Zalata, Ayman Z El-Samanoudy, Dalia Shaalan, Youssef El-Baiomy, Taymour Mostafa

**Affiliations:** 1 Department of Medical Biochemistry, Faculty of Medicine, Mansoura University, Mansoura, Egypt; 2 Department of Dermatology and Andrology, Faculty of Medicine, Mansoura University, Mansoura, Egypt; 3 Department of Andrology and Sexology, Faculty of Medicine, Cairo University, Cairo, Egypt

**Keywords:** Cell Phone, Spermatozoa, Electromagnetic Radiation, Sperm Motility

## Abstract

**Background:**

Use of cellular phones emitting radiofrequency electromagnetic field (RF-EMF) has been increased exponentially and become a part of everyday life. This study aimed to investigate the effects of *in vitro* RF-EMF exposure emitted from cellular phones on sperm motility index, sperm DNA fragmentation and seminal *clusterin (CLU)* gene expression.

**Materials and Methods:**

In this prospective study, a total of 124 semen samples were grouped into the following main categories: i. normozoospermia (N, n=26), ii. asthenozoospermia (A, n=32), iii. asthenoteratozoospermia (AT, n=31) and iv. oligoasthenoteratozoospermia (OAT, n=35). The same semen samples were then divided into two portions non-exposed and exposed samples to cell phone radiation for 1 hour. Before and immediately after exposure, both aliquots were subjected to different assessments for sperm motility, acrosin activity, sperm DNA fragmentation and *CLU* gene expression. Statistical differences were analyzed using paired t student test for comparisons between two sub-groups where p<0.05 was set as significant.

**Results:**

There was a significant decrease in sperm motility, sperm linear velocity, sperm linearity index, and sperm acrosin activity, whereas there was a significant increase in sperm DNA fragmentation percent, *CLU* gene expression and CLU protein levels in the exposed semen samples to RF-EMF compared with non-exposed samples in OAT>AT>A>N groups, respectively (p<0.05).

**Conclusion:**

Cell phone emissions have a negative impact on exposed sperm motility index, sperm acrosin activity, sperm DNA fragmentation and seminal *CLU* gene expression, especially in OAT cases.

## Introduction

Nowadays, cell phone technology is an integral part of everyday life, and its use will continue to grow as their providers proceed to offer more liberal services and newer, better products. Generally, a growing concern for possible adverse effects of cell phones on human health has evoked a flurry of scientific activity. Several studies have shown the association between human health and exposure to radiofrequency electromagnetic field ( RF-EMF ), emphasizing on clinical conditions as childhood leukemia, brain tumors, neurodegenerative diseases and genotoxicity (1). 

RF energy is a type of non-ionizing radiation, including electromagnetic radiation ( EMR ), produced by cell phones, but is not strong enough to cause ionization of atoms or molecules. Cellular phones emit low levels of RF in the micro-wave range while being used. Although high-level of RF causes adverse health effects through heating body tissues, exposure to low-level RF does not produce such effects. Several experimental studies demonstrated that exposure to electromagnetic or static magnetic fields had adverse effects on the reproductive system (2). 

De Iuliis et al. (3) demonstrated that RF-EMF in both the power density and frequency range of mobile phones enhances mitochondrial reactive oxygen species ( ROS ) generation in human spermatozoa that leads to decreased sperm motility and vitality, while stimulates DNA base adduct formation and, ultimately sperm DNA fragmentation. Kang et al. (4) showed that cell phone radiation may cause structural and functional injuries of the testis, alter semen parameters, and reduce epididymal sperm concentrations. In May 2011, the international agency for research on cancer ( IARC ) at World Health Organization ( WHO ) has categorized the RF-EMF from mobile phones, and from other devices that emit similar non-ionizing electromagnetic fields, as a group 2B ( possible ) human carcinogen (5). 

Sperm DNA fragmentation in the male germ line has been associated with impaired fertilization, poor embryonic development and high rates of miscarriage (6). Of course, the attention has been focused on the environmental and genetic factors that might be involved in the etiology of sperm DNA damage. One of these factors growing rapidly is the increased exposure to RF-EMF emitted from cell phones (7). 

*Clusterin (CLU)*, a 70-80 ku heterodimeric, disulfide-linked glycoprotein is over-expressed in a variety of tissues undergoing stress. *CLU* encoding clusterin appears to be a potential pathophysiologically gene having multiple functions related to apoptosis, inflammation, proliferation, and differentiation (8,9). 

This study aimed to assess the possible effects of *in vitro* RF-EMF exposure emitted from cell phones on sperm motility index, sperm DNA fragmentation and seminal *CLU* gene expression. 

## Materials and Methods

In this prospective study, semen samples were collected from 124 individuals presented to Mansoura University Hospital, Mansoura, Egypt, after Ethical Committee and Institutional Review Board approval with informed consent. Exclusion criteria were as follows: smoking, leukocytospermia, varicocele and abnormal karyotyping. Semen samples were collected by masturbation after an abstinence period of 4-5 days according to WHO guidelines (10). According to their semen analysis, they were grouped into the following main categories: normozoospermia ( N, n=26 ), asthenozoospermia ( A, n=32 ), asthenoteratozoospermia ( AT, n=31 ) and oligoasthenoteratozoospermia ( OAT, n=35 ). 

Each semen sample were divided into two portions, non-exposed ( control ) and exposed ( experimental ). Experimental semen samples were exposed to electromagnetic waves ( EMW ) emitted from a commercially available cellular phone ( 850 MHz frequency, maximum power <1 W, specific absorption rates 1.46 W/kg ) kept at 10 cm distance for 60 minutes. Unexposed semen aliquots were kept under the same conditions without RF-EMW exposure at room temperature to avoid the effect of temperature or reactive oxygen species ( ROS ) formation on semen parameters. After elapsed time, both aliquots were evaluated in terms of sperm motility, acrosin activity, sperm DNA fragmentation and seminal CLU gene expression before and immediately after exposure. 

### Sperm acrosin activity

It was assessed by gelatinolysis technique where gelatin-covered slides were prepared by spreading 20 μl of 5% gelatin ( Merck, Darmstadt, Germany ) in distilled water on the slides. The slides were airdried, stored at 4˚C overnight, fixed and washed in phosphate-buffered saline ( PBS, Sigma-Aldrich, St. Louis, MO, USA ). Purified spermatozoa were diluted 1:10 in PBS containing 15.7 mMol α-Dglucose ( Sigma-Aldrich, St. Louis, MO, USA ). Semen samples were incubated in a moist chamber at 37˚C for 2 hours. The halo diameter around any 10 sperms was measured in phase contrast using an eyepiece micrometer ( VWR, Radnor, PA, USA ). The halo formation rate was calculated/ slide as the percentage of spermatozoa showing a halo after evaluating 100 spermatozoa ( acrosin activity index=halo diameter×halo formation rate ) (11). 

### Sperm DNA fragmentation analysis

It was performed in fresh semen using flowcytometry ( DAKO-Cytomation, Glostrup, Denmark ) by a kit supplied by Coulter ( Beckman Coulter, Fulterton, CA, USA ) based on the fluorescence emission from individual sperm that was stained with propidium iodide ( PI ) and excited at 488 nm with an argon laser. Semen samples were diluted with PBS ( pH=7.4 ) to reach 2×10 ^6^ sperm/ ml where 50 μl were incubated with 100 μl lysing reagent for 15 seconds, and then 2 ml of PI were added and mixed. After staining, flowcytometry acquisition was performed where the intensity of its fluorescence emission corresponds to the DNA content. The analysis displays a constant and characteristic bimodal non-artifactual DNA pattern confirming the existence of two distinct populations. The main population is represented by a peak followed by a shoulder which is the marginal population representing a sperm group altered in the nuclear condensation (DNA damage), yielding unstable chromatin appearing more stainable. The Percentage of sperm cells with DNA damage is automatically calculated by the flowcytometer after acquisition of 5000 sperms(12). 

### CLU gene expression

Simultaneous isolation of total RNA and total proteins were done using Tri-Fast reagent kit ( PeqLab Biotechnologie GmbH, Germany ). The remaining DNA was digested using DNase I ( Sigma-Aldrich, St. Louis, MO, USA ). The concentration of isolated RNA was determined spectrophotometrically at optical density of 260 nm. Ten µl of each sample were added to 990 µl diethylpyrocarbonate ( DEPC )-treated water and quantified by measuring the absorbance at 260 nm as follows: RNA yield ( µg/ml )=A260×40×100 ( dilution factor ). Purity of RNA was assessed by gel electrophoresis through formaldehyde agarose gel electrophoresis ( Sigma-Aldrich, St. Louis, MO, USA ) and ethidium bromide staining ( Sigma-Aldrich, St. Louis, MO, USA) to show 2 sharp purified bands representing 28S and 18S ribosomal RNA. 

### RT-PCR for extracted RNA

Semi-quantitative reverse transcription-polymerase chain reaction ( RT-PCR ) was performed using ready-to-go RT-PCR beads for first cDNA synthesis and PCR reaction ( Amersham Biosciences, Piscataway, NJ, USA ) utilizing Moloney-murine leukemia virus reverse transcriptase ( M-MuLV RT ) and Taq polymerase to generate PCR product from RNA template. Each bead is optimized to allow the first strand cDNA synthesis and PCR reaction to proceed sequentially as a single tube, single step reaction. 

### Synthesis of cDNA

The following items were added to the tube containing the beads: 2 µl the first strand primer, 3 µl ( 30 pmol ) PCR gene-specific primer ( sense ), 3 µl ( 30 pmol ) PCR gene-specific primer ( anti-sense ), 25 µl total template RNA containing 1 µg and 17 µl DEPC-treated water to reach a total volume of 50 µl. One tube was prepared as a negative control reaction to test DNA contamination. The dehydrated bead ( without template and primers ) was incubated at 95˚C for 10 minutes to inactivate M-MuLV RT where 50 µl mineral oil was added to overlay the reaction. The reactions were transferred to the thermal cycler in order to be incubated at 40˚C for 30 minutes for synthesis of cDNA followed by incubation at 95˚C for 5 minutes to inactivate reverse transcriptase and to denature the template. The sequence of oligonucleotide primers of clusterin gene were designed from GenBank sequences 5΄-CTTGATGCCCTTCTCTCCGTA-3΄ ( sense ) and 5΄-AACGTCCGAGTCAGAAGTGTG-3΄ ( antisense ), located at nucleotides 684 to 704 and 1194 to 1214 of *CLU* cDNA. Thermal cycling reaction was performed as follows: 30 cycles of denaturation at 95˚C for 1 minute, annealing at 55˚C for 1 minute, extension at 72˚C for 1 minute and final extension at 72˚C for 10 minutes. The products were subjected to agarose gel electrophoresis using 2% agarose, stained with ethidium bromide, visualized via a light UV transilluminator, ( Clinx Science Instruments Co., Ltd, Shanghai, China ) and photographed. 

CLU protein was analyzed by Western blotting technique using rabbit anti-human CLU polyclonal unconjugated primary antibody against βtubulin as a control. Goat anti-rabbit IgG antibody conjugated to horseradish peroxidase ( HRP ) was used as secondary antibody. Colorimetric immunodetection of the protein was used as an enzyme substrate ( tetramethylbenzidine ) that reacted with the reporter enzyme ( HRP ) and precipitated into the conjugated antibodies. The bands on the membrane were digitally photographed and analyzed with Scion image release alpha 4.0.3.2 ( Scion Corporation, Frederick, MD, USA ) performing bands detection and conversion to peaks. Area under each peak was calculated in square pixels and used for quantification. CLU gene expression and CLU protein levels were determined by calculating the ratio between the square pixel values of the target bands in relation to the control bands. 

### Statistical analysis

It was performed using SPSS program version 17 ( SPSS Inc., Chicago, IL, USA ). The data were expressed as mean±standard deviation ( SD ). The statistical differences were analyzed using paired t-student test for comparison between two subgroups. P<0.05 was set as significant. 

## Results

**Fig.1:**
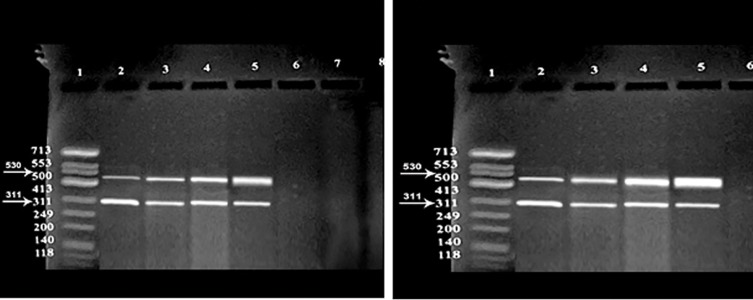
CLU gene expression of non-exposed groups (right) and exposed group (left) to mobile phone radiation. Lane1; DNA marker, Lane 2; N group, Lane 3; A group, Lane 4; AT group, Lane 5; OAT group, and Lane 6; Negative control, N; Normozoospermia, A; Asthenozoospermia, AT; Asthenoteratozoospermia, OAT; Oligoasthenoteratozoospermia and CLU; Clusterin.

**Fig.2:**
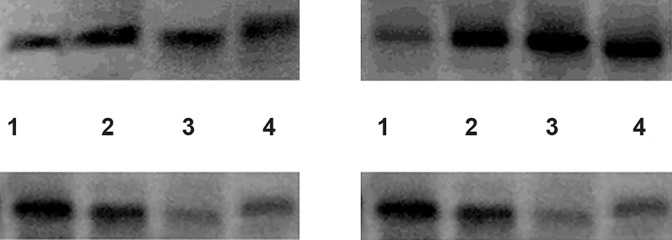
CLU protein expression by Western blotting (40 kd) in non-exposed groups (upper right) and exposed groups (upper left). Internal control; tubulin expression by Western blotting (50 kd) in the non-exposed groups (lower right) and exposed groups (lower left). Lane 1; N group, Lane 2; A group, Lane 3; AT group, Lane 4; OAT group, N; Normozoospermia, A; Asthenozoospermia, AT; Asthenoteratozoospermia, OAT; Oligoasthenoteratozoospermia and CLU; Clusterin.

The mean sperm concentration values in the N, A, AT and OAT groups were 54.34±5.0, 38.85±4.04, 23.52±8.94 and 8.00±3.77 ( 106/ml ), respectively. The mean percentage values of abnormal sperm belonging to the investigated groups ( A, AT and OAT groups ) were 11.42±2.61, 10.04±3.7, 30.80±7.22, 39.68±5.6, respectively. Sperm motility, sperm linear velocity, sperm linearity index and sperm acrosin activity were significantly decreased ( p<0.05 ). However, there is a significant increase in sperm DNA fragmentation percent, *CLU* gene expression and CLU protein levels in the exposed semen samples to RF-EMR compared with nonexposed samples in OAT>AT> A>N groups, respectively ( p<0.05 ). Semen samples of N group demonstrated a non-significant decrease in sperm motility, sperm linear velocity, sperm linearity index, and sperm acrosin activity, whereas demonstrated a significant increase in sperm DNA fragmentation percent, sperm *CLU* gene expression and CLU protein levels ( p<0.05 ) compared with the nonexposed samples (Table 1 ,Figs.1,2). 

**Table 1:** Estimated data in the exposed semen groups vs. non-exposed groups (mean±SD)


	N (n=26)		A (n=32)		AT (n=31)		OAT (n=35)	
	Non-exposed	Exposed	Non-exposed	Exposed	Non-exposed	Exposed	Non-exposed	Exposed

**Sperm motility %**	60.8±4.5	56.5±4.2	30.9± 5.4	26.5± 5.0^a^	23.3± 9.4	18.4±11.9^a^	17.7±10.9	12.7± 7.9^a^
**Sperm linear velocity %**	59.6±8.0	56.0±8.4	44.9± 14.7	39.1± 12.8^a^	25.5± 11.7	20.67±9.5^a^	23.8±13.6	16.6± 9.4^a^
**Sperm linearity index**	79.0±7.0	76.7±6.8	64.9± 10.2	56.5± 8.9^a^	66.0± 11.4	51.23±9.7^a^	58.5±15.8	41.3± 11.4^a^
**Sperm acrosin activity**	13.2±3.3	12.6±3.2	10.0± 2.4	8.3±2.0^a^	5.7±3.1	4.05±2.5^a^	2.5±2.6	1.8±1.9^a^
**CLU - RNA expression**	0.4±0.1	0.6±0.1^a^	0.8±0.3	1.5±0.6^a^	1.2±0.4	2.6± 0.8^a^	1.8±0.5	4.0±1.1^b^
**CLU -protein expression**	0.6±0.2	0.8±0.5^a^	0.8±0.2	1.4±0.4^a^	1.9±0.4	4.1± 0.8^b^	3.2±0.7	5.6±2.1^b^
**Sperm DNA fragmentation %**	11.5%	30.8%^b^	18.8%	56.3%^c^	29%	71.0%^c^	40.0%	80%^c^


^a^; Significant difference compared with unexposed semen samples (p<0.05). ^b^; Significant difference compared with unexposed semen samples (p<0.01), ^c^; Significant difference compared with unexposed semen samples (p<0.001), N; Normozoospermia, A; Asthenozoospermia, AT; Asthenoteratozoospermia, OAT; Oligoasthenoteratozoospermia and CLU; Clusterin.

## Discussion

In the current study, semen exposure to RFEMF led to a significant decrease in sperm motility compared to non-exposed semen samples. Previously, Fejes et al. (7) in an epidemiological study have pointed negative correlation between cell phone use and various attributes of semen quality, particularly sperm motility. This was followed by an experimental study involving exposure of male mice to RF-EMF that revealed a significant impact on the integrity of both the mitochondrial and nuclear genomes (13). Kilgallon and Simmons (14) demonstrated that storage of mobile phones close to the testes can decrease semen quality. Similarly, Erogul et al. (15) reported decreased sperm motility in the samples exposed to 900 MHz cell phone for 5 minutes where non-progressive and immotile sperm populations were increased after exposure. Agarwal et al. (16) confirmed such negative impact on semen quality correlating defects in sperm count, motility, viability and normal morphology, with longer duration of usage independent of the initial semen quality. Agarwal et al. (17) added that exposed spermatozoa to mobile phone radiation for 1 hour leads to significant declines in sperm motility and vitality associated with increased cellular ROS generation coupled with decreased ROS-total antioxidant capacity score. 

Such a significant decline in sperm motility was explained by intrinsic ROS generation reinforced with a significant increase in sperm DNA fragmentation in the exposed semen samples compared to the unexposed one in *in vitro* culture (18). Several lines of evidence suggested that oxidative stress ( OS ) plays a key role in the underlying etiology. Spermatozoa are sensitive to such stress as they possess limited endogenous antioxidant protection while presenting abundant substrates for free radical attack in the form of unsaturated fatty acids and DNA (19). Moskovtsev et al. (20) showed that EMF of cell phones may cause DNA breakage in spermatozoa in a low-frequency EMF that is likely due to stimulation of spermatozoa’s plasma membrane redox system by ROS production. De Iuliis et al. (3) added that RF-EMF in both the power density and frequency range of mobile phones enhances mitochondrial ROS generation by human spermatozoa, decreasing its motility and vitality while stimulating DNA base adduct formation and, ultimately sperm DNA fragmentation. 

It has been suggested that spermatozoa are particularly vulnerable to the induction of OS by RF-EMF, while a decrease in sperm motility and viability is expected to be linked to concentration of superoxide anion in semen that can oxidize sperm membrane phospholipids. In addition, these reported effects could be attributed to thermal insult induced by RF exposure (18). Aitken et al. (13) observed a significant impact on the integrity of both the mitochondrial and nuclear genomes after exposure of male mice to RF-EMF. In contrast, McNamee et al. (21), Tice et al. (22) and Stronati et al. (23) demonstrated non-significant DNA damage in human cells exposed to RF-EMF. 

De Iuliis et al. (3) suggested that excess exposure to RF-EMF emitted from mobile phones is one of the key environmental factors involved in the stimulation of sperm mitochondria that results in producing high levels of ROS. Moreover, such stress is known to induce functional and structural lesions including loss of sperm motility mediated by peroxidative damage to the sperm plasma membrane, as well as to form DNA base adducts in the sperm nucleus leading to DNA fragmentation (24). Agarwal et al. (25) concluded that DNA damage due to EMW is significant, but this damage may be the result of cumulative effect of repeated exposure, not revealed after short term exposures. 

Exposure to emitted radiation from mobile phones was demonstrated to have an up-regulation of both *CLU* mRNA and its full length protein in infertile semen samples compared with the normozoospermic samples. Strocchi et al. (26) hypothesized that increased levels of *CLU* mRNA in morphologically normal cells were due to cellular stress response in which cells attempt to protect themselves from local stress conditions. Therefore, increased *CLU* expression could be explained by the physiological defense to reduce cell damage and to maintain cell viability during periods of exposure exerted through *CLU*, ability to act as a scavenger. Trougakos and Gonos (8) proposed that *CLU* with its antioxidant properties is capable of protecting cells from apoptosis induced by ROS. Strocchi et al. (27) supported the notion that an increase in *CLU* expression may be a physiological defense mounted to reduce cell damage and to maintain cell viability during periods of increased OS. 

Therefore, increased *CLU* expression was associated in parallel with increased sperm DNA fragmentation and decreased sperm acrosin activity being triggered by OS (28). It is suggested that OS plays a key role in the underlying sperm DNA fragmentation as well as acrosin activity. When ROS production by the sperm’ mitochondria is excessive, the gamete’s limited endogenous antioxidant defenses are rapidly overwhelmed, and oxidative damage is induced, leading to peroxidation of the sperm acrosomal membrane and diminished acrosin activity (29,32). 

A point of limitation in this study is the inability to assess the effects of multiple exposures in addition to reversibility effects to know whether sperm affections are time related or not that is needed for further work. Also, a future study is suggested to be conducted on the comparison of the effects of RF radiation between iPads and cell phones on sperm motility, sperm DNA fragmentation and seminal *CLU* gene expression. 

## Conclusion

Cell phone emissions have a negative impact on sperm motility, sperm acrosin activity, sperm DNA fragmentation and *CLU* gene expression, especially in OAT cases. 
